# Stress-induced HPA activation in virtual navigation and spatial attention performance

**DOI:** 10.1186/s12868-022-00722-y

**Published:** 2022-06-28

**Authors:** Anthony E. Richardson, Melissa M. VanderKaay Tomasulo

**Affiliations:** Department of Psychology, Saint Michael’s College, One Winooski Park, Colchester, VT 05439 USA

**Keywords:** Acute stress, Salivary cortisol, Cardiovascular reactivity, Socially evaluated cold pressor test, Useful field of View, Virtual navigation, Spatial learning

## Abstract

**Background:**

Previous research has shown that spatial performance (e.g. navigation, visuospatial memory, attention) can be influenced by acute stress; however, studies have produced mixed findings sometimes showing an improvement after stress, other times showing impairment or no overall effect. Some of these discrepancies may be related to: the type of stress system activated by the stressor (sympathetic adrenal medulla [SAM] or hypothalamic-pituitary-adrenocortical [HPA]); whether cortisol responders vs. nonresponders are analyzed subsequent to main effects; and sex differences in stress responses. In the present study, we examine the influence of HPA activation from an acute laboratory stressor (Socially Evaluated Cold Pressor test [SECPT]) on performance during two spatial tasks: Useful Field of View (UFOV; a measure of spatial attention) and virtual reality (VR) navigation. We assigned 31 males and 30 females to either the SECPT or a Non-Stress condition prior to the two spatial tasks. Cardiovascular measures including heart rate and blood pressure, and salivary cortisol biosamples were obtained at specific time points.

**Results:**

Participants in the Stress condition showed increases in heart rate, systolic and diastolic blood pressure indicating sympathetic adrenal medulla (SAM) axis activation. Stress also led to increases in salivary cortisol, suggesting hypothalamic-pituitary-adrenocortical (HPA) activation. Stress did not influence overall performance in the spatial attention UFOV or the VR navigation task. However, a sex difference in spatial attention was detected when participants were divided into Stress-cortisol responders and non-responders in the UFOV task. Male Stress-cortisol responders (*n* = 9) showed better UFOV accuracy than female Stress-cortisol responders (*n* = 6); no sex differences were found among the Non-Stress control group. Furthermore, for females in the stress condition (*n* = 14), higher cortisol responses were associated with lower spatial attention performance.

**Conclusions:**

Socially Evaluated Cold Pressor stress resulted in no change in speed or accuracy in a VR navigation task. For the spatial attention task, the SECPT led to a sex difference among Stress-cortisol responders with males showing improved accuracy over females. The relationship between HPA activation and prefrontal cortex activity may be necessary to understand sex differences in spatial attention performance.

## Background

In acute stressful situations such as navigating through traffic, participating in competitive sports, and public speaking, people may need to attend to the environment, encode, and retrieve memories or make decisions. During these periods of psychological stress, two physiological systems are activated: (1) the fast acting sympathetic adrenal medulla (SAM) axis, leading to the release of catecholamines such as epinephrine/norepinephrine, which stimulate heart rate and blood pressure, and (2) the slower hypothalamic pituitary adrenal (HPA) axis which results in the release of the glucocorticoids such as cortisol. Previous experience with stressors can mediate the HPA response and subsequent release of cortisol [1]. Activation of these biological stress systems may become influential during activities that require attention, learning, and memory [2, 3] and researchers have systematically investigated the complex interaction between psychological stress and these processes.

While much of the research investigating learning and memory in humans has focused on verbal performance, such as digit span or memory for word lists, researchers have also examined the dynamic relationship between stress and spatial cognition, such as virtual navigation and visuospatial memory. For example, Duncko et al. [4] administered a one-minute cold pressor test (a physical stressor via hand exposure to ice water) prior to a virtual navigation Morris water task [5] task within males. Participants in the stress condition showed increased SAM activation, as indicated by an increase in heart rate and skin conductance but no cortisol response, and also showed improved performance on the virtual navigation task. A similar pattern of SAM activation without a cortisol response was induced in a study from our lab using a different timed spatial stressor (Star Mirror Trace Task) [6]. In contrast to Duncko, stress here resulted in slower performance in both males and females in large scale virtual navigation and in a spatial perspective taking task, which involved making spatial judgments from different imagined orientations.

Psychosocial stressors, such as the Trier Social Stress Task (TSST) [7] have also been utilized as they produce more reliable and strong activation of both SAM and HPA [8] due to their socially evaluative nature. Klopp et al. [9] replicated Duncko et al. [4], but used the TSST instead of the physical cold pressor stressor. In this case, stress resulted in increases in salivary alpha amylase and cortisol, indicating activation of both SAM and the HPA axis, respectively. However, despite activation of both stress systems and contrary to Duncko et al. [4], stress resulted in no change in navigation performance. Schwabe et al. [10] also showed that TSST stress resulted in elevated cortisol and heart rate, but had no effect on single target location learning in 3D models. This would seem to further support the view that HPA activation does not lead to changes in spatial performance. Although most studies assess performance through response time and accuracy measures, acute stress may also have more subtle effects on spatial [9, 10] performance. Interestingly, even though Schwabe et al. [10] found no overall differences in accuracy, participants in the stress condition shifted from a spatial strategy to a stimulus response learning strategy. The authors suggested that stress hormones such as catecholamines and glucocorticoids may be modulating the use of dorsal striatum-based (stimulus response) and hippocampus-based (spatial) memory systems. Other studies have also shown strategy shifts in virtual reality navigation after mental arithmetic stress (Paced Auditory Serial Addition Task [PASAT]) [11] and time-pressure induced stress [12]. Taken together, evidence for the effect of acute stress on spatial navigation is inconclusive, with both impairment and enhancement effects after SAM activation and a lack of performance differences following HPA involvement, although previous studies did reveal stress-dependent changes in task strategies [9, 10].

Studies examining the influence of acute stress on visuospatial memory performance reveal a different pattern of results than the virtual navigation literature. For example, in studies investigating spatial working memory, participants completed a spatial 3-back task where they were presented with a stream of letters and judged whether the location of a letter matched the location of a letter presented 3 trials previously. Three studies demonstrated impaired performance in spatial working memory *n-*back after shock threat [13, 14] or TSST stress [15]. Notably, these studies resulted in SAM activation but presented no cortisol increase. In contrast, stress studies resulting in HPA activation demonstrated that increases in cortisol are associated with improvements in visuospatial map memory [16], visual working memory for faces and scenes [17], and visuospatial change detection [18]. On the contrary, increased cortisol responses have also been associated with no change in visuospatial memory performance on the Rey-Osterrieth Complex Figure task (ROCF) [19–21].

While moderate levels of acute stress have been commonly examined in laboratory settings, due to ethical constraints, only a handful of intense stress studies have been conducted. These studies consistently show that extreme stress is associated with impairment in visuo-spatial tasks. In a field experiment with a special military population, Morgan et al. [22] found that survival in a mock prisoner of war (POW) camp resulted in visuospatial memory impairment as assessed by the ROCF task [23] for both males and females. Physiological measurements (e.g. catecholamines, glucocorticoids) were not obtained and thus HPA activation was uncertain; however, a comparable study conducted by Taverniers et al. [24] found similar results. They demonstrated that POW stress produced elevated cortisol and visuospatial impairment with the ROCF and a decrease in working memory performance as assessed by a digit span task [25] in a sample of male Special Forces candidates. Furthermore, cortisol levels were negatively correlated with ROCF scores in that greater HPA activity was associated with worse performance. High stress due to parachute jump training is also associated with lower performance in a visuo-spatial path learning task [24]. Examining similar performance measures, but using a more moderate stressor, may produce different results.

Hoffman and Al’absi [21] found no effect of public speaking induced stress on visuo-spatial memory (ROCF) and digit span despite reporting elevated cortisol levels. The discrepancy between the Hoffman and Al’absi study and the aforementioned military population studies may be the result of an inverted U-curve relationship between HPA activity and performance. Small amounts of cortisol secretion might show improved performance up to an optimal level, but higher levels of stress then led to diminished abilities [26, 27]. The relationship between acute stress and spatial cognition is clearly complex; however, one factor that might contribute to the inconsistency among previous research is that each study examined stress at different points along the inverted U-curve.

As with spatial navigation and visuospatial memory, studies examining attention have shown inconsistent results with some showing facilitation and other impairments after exposure to acute stress. For example, Stroop-type selective attention performance increased after exposure to noise, time pressure, and social evaluation [28, 29] with the implication that stress exposure may improve the ability to ignore irrelevant information. Including a task that leads to cortisol release via HPA activation, Schwabe and Wolf [30] used a Socially Evaluated Cold Pressor Test (SECPT) [31] to study the attentional blink [32] in male participants. Attentional blink refers to the impaired processing of a second target when it is presented within a few hundred milliseconds after a first target. Acute stress resulted in improved accuracy, and high cortisol responders showed improved attention through a smaller attentional blink.

However, incongruent results were found by Sänger et al. [33] when using the SECPT to induce stress in males before a selective attention task. Participants in their study were required to detect luminance changes while ignoring distracting and irrelevant orientation changes. The SECPT resulted in both increased salivary alpha amylase (an indicator of SAM activation) and cortisol, and higher error rates in attentional selection, suggesting that stress increases distractibility. Focused attention in simple and choice reaction time tasks were also impaired in male only participants after TSST stress [15]. Similarly, within baggage screening novices, impaired detection of threat objects viewed through an X-ray task after TSST stress has been observed [34].

More studies are needed to further clarify the effects that SAM and HPA activation can have on spatial performance. In the present study, our first goal was to examine the effect of an acute psychosocial stressor (SECPT) on spatial learning through VR navigation (indexed by orientation accuracy and response time measures after traversing through a virtual environment). Based on previous research [9–11], we predicted our stressor would have no overall effect on measures of spatial navigation; however, dividing the stress group into cortisol responders and non-responders might reveal performance differences [35, 36]. Our second goal was to examine the influence of acute stress on spatial attention (indexed by accuracy on the Useful Field of View task). The UFOV is a commonly used measure of selective attention in which targets are presented rapidly and must be detected at different eccentricities from the center of the screen. Previous research examining the relationship between stress and spatial attention is inconsistent [33, 34, 36], and the UFOV has never been investigated with respect to stress. Cardiovascular measurements were collected to assess SAM activation throughout the study session and salivary cortisol measurements were obtained to assess HPA activation and allow for further cortisol responder analysis.

## Results

### SAM activation

**Fig. 1:**
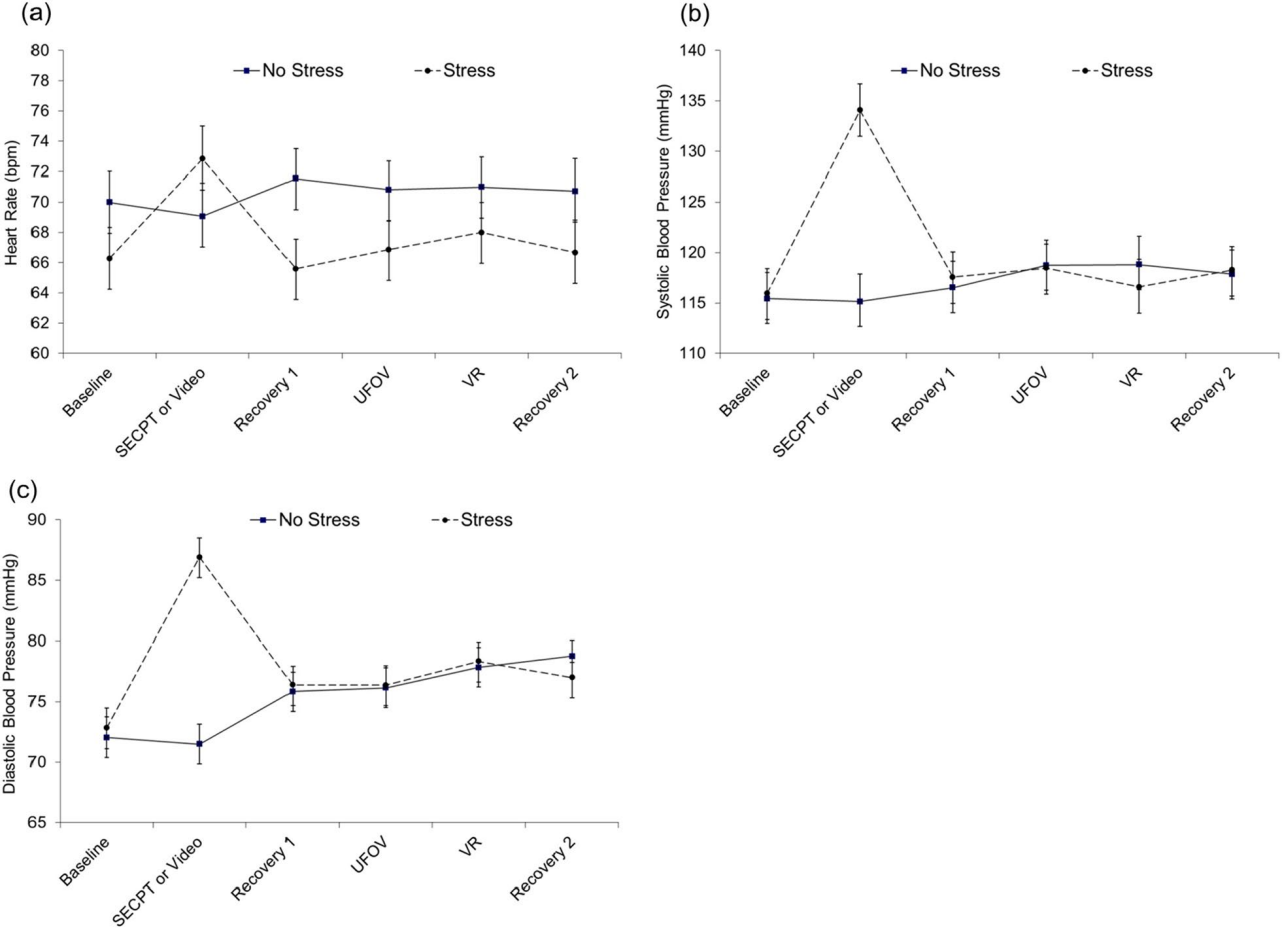
Mean (± SEM) for (**a**) Heart Rate, (**b**) Systolic Blood Pressure, and (**c**) Diastolic Blood Pressure values for participants in the No Stress (Video) and Stress (SECPT) conditions at periods throughout the experiment

A series of 6 × 2 × 2 mixed model ANOVAs with Task (Baseline, SECPT or control Video, Recovery 1, UFOV, VR, and Recovery 2) as the within subjects factor and Stress condition (Stress or Non-Stress) and Sex (males or females) as the between subjects factors were conducted for HR, SBP, and DBP, as dependent measures. Task main effects were found for HR, *F*(5, 270) = 6.81, *p* < 0.001, *η*_*p*_^*2*^ = 0.11, SBP, *F*(5, 270) = 13.72, *p* < 0.001, *η*_*p*_^*2*^ = 0.20, and DBP, *F*(5, 270) = 13.18, *p* < 0.001, *η*_*p*_^*2*^ = 0.20 (see Fig. 1). Sex main effects indicated that females had overall greater HR as compared to males, *F*(1, 54) = 6.10, *p* = 0.017, *η*_*p*_^*2*^ = 0.10, while males had overall greater SBP than females, *F*(1, 54) = 11.68, *p* = 0.001, *η*_*p*_^*2*^ = 0.18. No main effect for Stress condition was demonstrated for any of the cardiovascular measures.

Significant Task by Stress interactions were demonstrated for HR, *F*(5, 270) = 19.27, *p* < 0.001, *η*_*p*_^*2*^ = 0.26, SBP, *F*(5, 270) = 21.94, *p* < 0.001, *η*_*p*_^*2*^ = 0.29, and DBP, *F*(5, 270) = 23.56, *p* < 0.001, *η*_*p*_^*2*^ = 0.30 (see Fig. 1). When Bonferroni adjustments to *p* values were applied to post-hoc tests, participants in the Stress condition had greater HR, *F*(1, 28) = 33.18, *p* < 0.001, *η*_*p*_^*2*^ = 0.54, and SBP, *F*(1, 29) = 78.42, *p* < 0.001, *η*_*p*_^*2*^ = 0.73, and DBP, *F*(1, 29) = 67.90, *p* < 0.001, *η*_*p*_^*2*^ = 0.70, during the SECPT as compared to Baseline; thus indicating that our stressor was effective at producing hemodynamic changes. Between subjects effect comparisons showed that SBP, *t*(58) = 4.64, *p* < 0.001, *d =* 1.20, and DBP, *t*(58) = 6.55, *p* < 0.001, *d =* 1.69, were greater for participants in the Stress condition as compared to the Non-Stress condition during the SECPT or control Video period.

### HPA activation

A 4 × 2 × 2 mixed model ANOVA with Cortisol Time (Baseline, post-UFOV, post-VR, post-Recovery 2) as the within subjects factor and Stress (stress or non-stress) condition and Sex (males or females) as the between subjects factors was conducted. Because cortisol values were positively skewed, the statistical analysis was performed with log transformed cortisol values.

Main effects were found for Cortisol Time, *F*(3, 162) = 9.90, *p* < 0.001, *η*_*p*_^*2*^ = 0.16. A Stress condition main effect was also demonstrated, *F*(1, 54) = 8.49, *p* = 0.005, *η*_*p*_^*2*^ = 0.14, such that cortisol levels were greater during the Stress condition as compared to the Non-Stress condition. A significant Cortisol Time by Stress interaction was also demonstrated, *F*(3, 162) = 12.18, *p* < 0.001, *η*_*p*_^*2*^ = 0.18. When Bonferonni adjustments to the *p* values were applied to post-hoc tests, for participants in the Stress condition Cortisol values were greater post-UFOV as compared to Baseline, *F*(1, 28) = 14.69, *p* < 0.001, *η*_*p*_^*2*^ = 0.34; thus indicating that our stressor was effective at producing a cortisol response. For participants in the Non-Stress condition, Cortisol values were lower post-VR, *F*(1, 28) = 22.14, *p* < 0.001, *η*_*p*_^*2*^ = 0.44, and post-Recovery 2, *F*(1, 28) = 14.45, *p* < 0.001, *η*_*p*_^*2*^ = 0.34, as compared to Baseline. Between subjects effect comparisons showed that post-UFOV Cortisol, *t*(57) = 3.86, *p* < 0.001, *d* = 1.01, post-VR Cortisol, *t*(57) = 3.61, *p* < 0.001, *d* = 0.94, and post-Recovery, *t*(56) = 2.86, *p* = 0.006, *d* = 0.75, were greater for participants in the Stress condition as compared to the Non-Stress condition, thus showing that cortisol remained elevated throughout the task periods for the Stress condition. A significant Sex by Stress interaction was also found, *F*(1, 54) = 4.52, *p* =  0.038, *η*_*p*_^*2*^ = 0.08. Between subjects effect comparisons demonstrated that males in the Stress condition had greater cortisol values than males in the Non-Stress condition, *F*(1, 28) = 10.93, *p* = 0.003, *η*_*p*_^*2*^ = 0.28. For females, cortisol values were not significantly different across stress conditions, *F*(1, 26) = 0.39, *p* = 0.54, *η*_*p*_^*2*^ = 0.02.

**Fig. 2:**
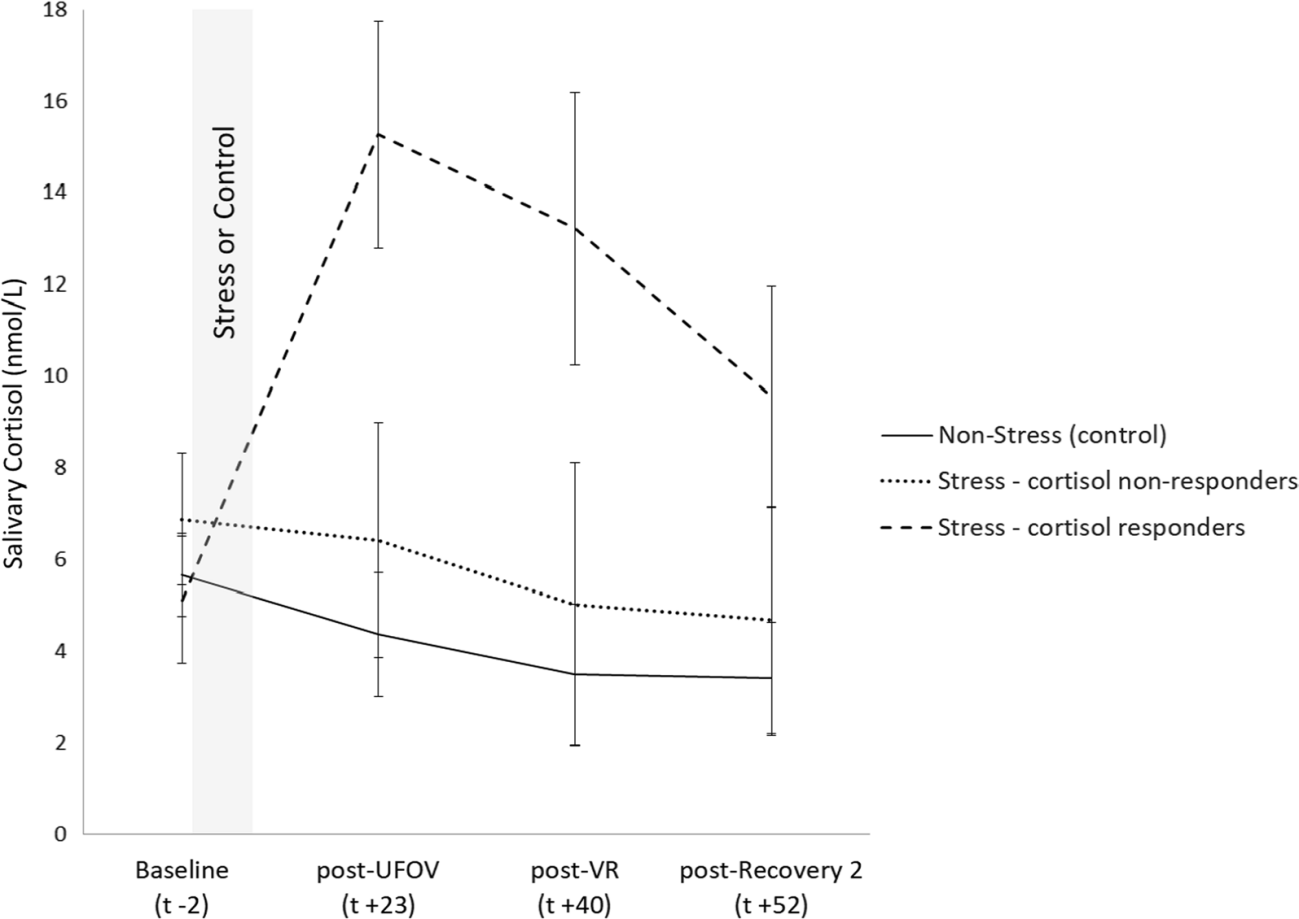
Mean salivary cortisol values (± SEM) for Baseline, post-UFOV, post-VR, and post-Recovery 2 by Stress-Cortisol responder type (responders vs. non-responders) and Non-Stress condition. The first cortisol sample occurred 2 min before onset of the Socially Evaluative Cold Pressor Test (SECPT) or Non-Stress (control Video) t-2; subsequent collection times are approximate

Because of the high variability in cortisol response across individuals (see Fig. 2), participants in the Stress condition were divided into cortisol responders (increases greater than 1.5 nmol/L) and non-responders (less than 1.5 nmol/L) in accordance with criteria established by Miller et al. [37]. This resulted in a group of 15 cortisol responders (9 males, 6 females) and a group of 14 cortisol non-responders (6 males, 8 females). A 2 × 2 Chi-square analysis between Sex (males or females) and cortisol responder status (responders, non-responders) was conducted to test for independence between the variables, χ^2^(1, *n* = 29) = 0.85, *p* = 0.36. There is no evidence that cortisol responder status depends upon sex.

### Useful field of view (UFOV)

A 2 × 2 factorial ANOVA with Stress (Stress or Non-Stress) condition and Sex (males or females) as between subjects factors was conducted with UFOV accuracy as the dependent measure. Participants in the Stress condition (*M* = 0.58, SD = 0.25) did not differ in accuracy from those in the Non-Stress condition (*M* = 0.63, SD = 0.23), *F*(1, 57) = 0.624, *p* = 0.433, *η*_*p*_^*2*^ = 0.01, and males (M = 0.56, SD = 0.231) did not differ from females (M = 0.56, SD = 0.24), *F*(1,57) = 2.21, *p* = 0.143, *η*_*p*_^*2*^ = 0.04.

**Fig. 3:**
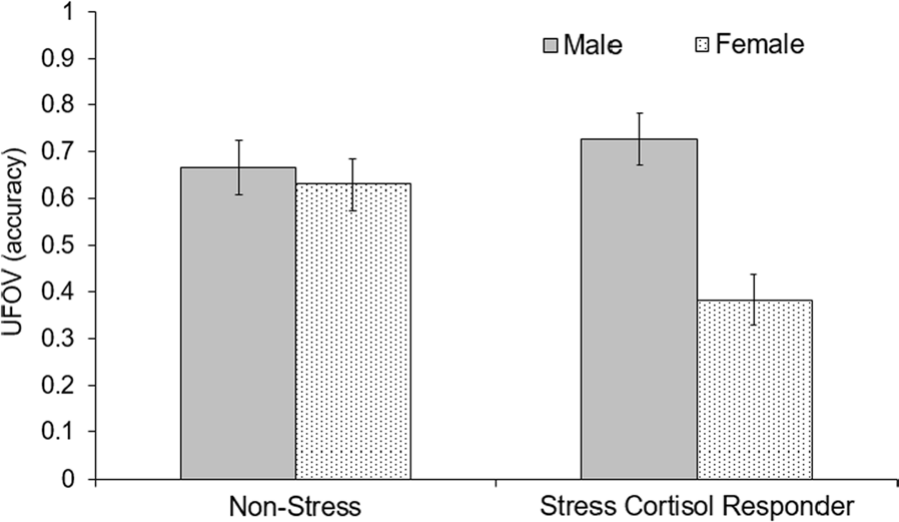
Responder (Stress-cortisol responder, Non-Stress condition) by Sex interaction for UFOV accuracy (Mean ± SEM)

A 2 × 2 factorial ANOVA with Responder (Stress-cortisol responder or Non-Stress condition) and Sex (males or females ) as between subjects factors was conducted with UFOV accuracy. A main effect for Sex indicated that males (*M* = 0.69, SD = 0.20) had greater UFOV accuracy as compared to females (*M* = 0.56, SD = 0.24), *F*(1, 40) = 7.74, *p* = 0.008, *η*_*p*_^*2*^ = 0.16. A Stress condition (Stress-cortisol responder or Non-Stress) by Sex interaction was demonstrated, *F*(1, 40) = 5.04, *p* = 0.03, *η*_*p*_^*2*^ = 0.11 (see Fig. 3). No Sex differences were demonstrated in the Non-Stress condition, *t*(27) = 0.46, *p* = 0.65; however, within Stress-cortisol responders, males had greater accuracy than females, *t*(13) = 3.12, *p* = 0.008, *d* = 1.64. Additionally, males in the Non-Stress condition did not differ in accuracy from male Stress-cortisol responders, *t*(22) = 0.69, *p* = 0.50. For females however, Stress-cortisol responders were less accurate than females in the Non-Stress condition, *t*(18) = 2.30, *p* = 0.034, *d* = 1.12.

**Fig. 4:**
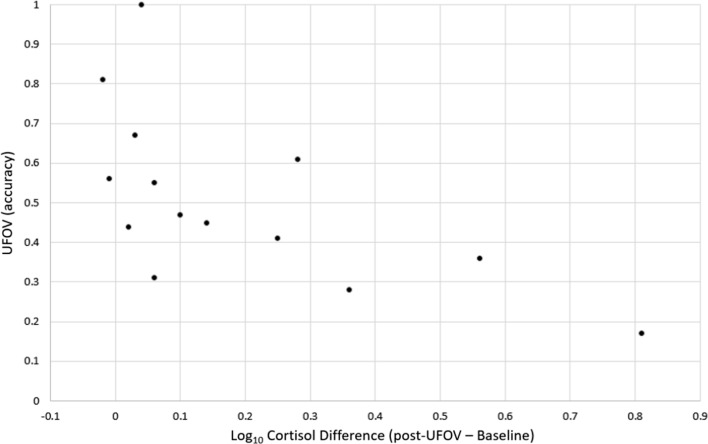
Female participants’ (*n* = 14) change in log salivary cortisol concentration between peak levels (post-UFOV; t +23) subtracted from Baseline levels (t **–**2), *r*(12) = −0.63, *p* =  0.016

Correlational analyses conducted between Cortisol change scores and UFOV accuracy also showed a similar relationship. Change scores were calculated by subtracting the peak cortisol values (log-transformed Cortisol post-UFOV values) from values obtained during Baseline (also log-transformed). Among females in the stress condition, a greater cortisol response was associated with lower accuracy scores, *r*(12) = −0.63, *p* = 0.016 (see Fig. 4). Males in the stress condition showed an opposite relationship in that greater cortisol response was associated with greater accuracy scores; however, this relationship was not significant, *r*(13) = 0.33, *p* = 0.23. On the possibility that performance is curvilinearly related to cortisol response, we also tested for quadratic relationships between cortisol change scores and UFOV accuracy; however, no significant relationship was found.

### Virtual reality navigation performance

A 2 × 2 factorial ANOVA with Stress (Stress or Non-Stress) condition and Sex (males or females) as between subjects factors was conducted with VR pointing error (the deviation between the observed and correct response in degrees) and VR response time as dependent measures. Response time was log transformed to correct for positive skew. For VR pointing error, participants in the Stress condition (*M* = 23.13, SD = 10.54) did not differ from those in the Non-Stress condition (*M* = 24.36, SD = 11.90), *F*(1, 56) = 0.181, *p* = 0.67, *η*_*p*_^*2*^ = 0.003. Participants in the Stress condition (*M* = 0.75, SD = 0.14) also did not differ from those in the Non-Stress condition (*M* = 0.73, SD = 0.15) for VR log response time, *F*(1, 56) = 0.264, *p* = 0.61, *η*_*p*_^*2*^ = 0.005.

The effect of stress-induced HPA activation on VR performance was also investigated. Similarly to our strategy when conducting UFOV analyses, participants in the Stress condition were divided into cortisol responders (increases greater than 1.5 nmol/L) and non-responders (less than 1.5 nmol/L). A 2 × 2 factorial ANOVA with Stress (Stress-cortisol responder or Non-Stress) and Sex (males or females) as between subjects factors was conducted with VR pointing error and VR log response time as the dependent measures. No main effects or interactions were found for either variable.

## Discussion

In this study, we examined the effects of an acute Socially Evaluative Cold Pressor (SECPT) stressor on two subsequently performed spatial tasks – Useful Field of View (UFOV) spatial attention task and VR navigation. To our knowledge, this was the first study to examine UFOV spatial attention performance after exposure to acute laboratory stress.

Similar to previous research [31, 38, 39] our study demonstrated that acute stress resulted in increased cardiovascular measurements as compared to Baseline levels; thus indicating that our stress manipulation was effective at producing sympathetic adrenal medulla (SAM) activation. As expected for the Stress condition, peak elevations occurred during the SECPT stressor, while participants in the Non-Stress condition did not show cardiovascular increases during the nature video presentation. Also consistent with previous research [40, 41], males in our study had greater SBP overall, while females had greater HR. Similar to Schwabe et al. [31], the SECPT also resulted in hypothalamic pituitary adrenal (HPA) activation, such that participants in the Stress condition had greater salivary cortisol values post stress, which returned to near baseline levels at the conclusion of the study. Participants in the Non-Stress condition did not have increases in cortisol and instead showed a steady decrease throughout the experiment. Between subjects comparisons demonstrated that except for during Baseline, participants in the Stress condition had consistently greater cortisol values at all time periods as compared to the Non-Stress condition. Males in the Stress condition also demonstrated greater cortisol activation than females, which is consistent with previous research [42, 43]. Based on these findings, the authors therefore believe that the SECPT was an effective stressor at producing both SAM and HPA activation.

Overall, stress exposure did not influence spatial attention UFOV accuracy, and there were no sex differences in performance. Previous studies examining the UFOV without the influence of stress have found mixed results, with some studies reporting no sex differences in performance on the UFOV [44, 45], which is consistent with our control condition and another reporting a male advantage [46], which is the pattern demonstrated among our cortisol responders. To account for substantial variability in HPA reactivity, for the analysis of our spatial tasks, we divided our participants into Stress Responders and Stress Non-responders. Subsequently, we demonstrated that male cortisol responders were more accurate than female cortisol responders on the UFOV task (*d* = 1.64) and that among females, increases in a cortisol response were associated with lower UFOV accuracy (*r* = −.63). Since our work is the first study investigating stress in relation to the UFOV, consideration of underlying mechanisms remains speculative. One explanation for this interaction may be connected to the relationship between cortisol and the executive control network, which involves the prefrontal cortex (PFC) [47]. The PFC is an area with a high density of glucocorticoid (i.e. cortisol) receptors [48], and the UFOV is associated with PFC functioning [49, 50]. However, the relationship between stress and PFC activity is unclear with some studies showing acute stress associated with an increase in PFC activation [51–53] and others with a decrease [47, 54]. Notably, one all-female study showed that stress led to decreases in PFC activation [55], which is consistent with the diminishment in UFOV accuracy in our study among female cortisol responders. Moreover, for our study, the UFOV task occurred before expected peak cortisol levels, which is further in line with a reduction in PFC functioning. Other studies have also shown similar sex differences after HPA activation in PFC-related tasks. In the *n*-back working memory task, Schoofs et al. [56] found that HPA activation from acute stress resulted in enhanced performance among men, but impaired performance among women. In the PFC-related Wisconsin Card Sorting Test of executive function, high cortisol levels were associated with more errors in women and fewer errors in men [57].

**Fig. 5:**
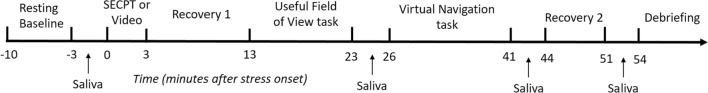
Timing of Tasks and Measurements

Our findings indicate that stress exposure did not result in a change in VR accuracy or response time, which is consistent with other virtual navigation research involving stressors with a cortisol response [9, 10, 20, 35]. Two studies did demonstrate an influence of stress on navigation performance [4, 6]; however, these studies differed in that their stressors involved SAM activation only. In our study, the VR task was always presented after the UFOV task, so that we could measure peak post-stress cortisol samples at the same time throughout the study session (see Fig. 5). Since the VR task was presented second, the influence of SAM would have likely diminished (occurring 25–40 min post stress). However, cortisol levels were high during the VR task, which likely influenced changes in performance. Previous studies indicate that cortisol levels peak between 20 and 35 min post-stress onset [10, 43]. For our study, these peaks occurred between the UFOV and VR tasks (see Fig. 2), which was approximately 23 min post-stress onset. The UFOV task however, would have been influenced by both a rising cortisol and an abating catecholamine-driven stress response.

According to Hermans et al. [47] different phases of the stress response are associated with changes in both neuroendocrine and executive control resources (see [47]; Fig. 1). The initial phase of stress (SAM activation) results in reduced PFC activation and less resources allocated to executive control. Although PFC activation was not measured in our study, this pattern would be consistent with our diminished performance among females in the UFOV task. In the subsequent phase of the stress response, catecholamines decrease while cortisol levels rise. Here one might expect a reduced attenuation or no effect, which was what we observed in our study during the VR task. If an even longer time period elapsed (beginning around one-hour after stress onset), later genomic cortisol effects may contribute to a reversal in the initial effects from stress, leading to an upregulation of executive control resources involving the PFC (see [58] for an extensive review). During this phase one might expect an improvement in spatial attention or navigation; however, to date no study has examined performance on such tasks in this longer time frame.

We believe that the specific type of stressor prior to spatial challenges may be an important factor in explaining these differential findings. Additionally, as previously stated the relationship between the central nervous system and cortisol response can be affected by the type of stressor one is exposed to. Stress researchers categorize stressors in many ways including, but not limited to: chronicity, predictability, controllability, or evaluative threat [1]; physical versus psychological [8]; and reactive versus anticipatory [59]. Some overlap among these definitions may occur; for example, our study may be categorized as both reactive and anticipatory. Our study utilized a stressor (SECPT) which encompasses both elements of pain (reactive) and anticipatory (socially evaluated threat) and may thus engage multiple brain regions. Specifically, reactive stressors have been found to affect the brainstem and hypothalamus while anticipatory stressors have been found to involve the hippocampus, amygdala, and PFC [59].

## Limitations and future directions

There were several possible limitations that may have influenced our results. Our UFOV accuracy finding was based on a Sex by Stress-cortisol responder interaction with a sample of *n* = 29. Low power from small sample size increases uncertainty in the magnitude of effect size. In this interaction, partial eta = 0.11 (which would represent a medium to large effect [60]) might be inflated. Low power also may have affected the analysis for the independence between Sex and Responser status. Based upon the non-significant chi square analysis, we have not demonstrated that Responder status depends upon Sex; however, this result might be a Type 2 error.

We assessed accuracy and reaction time in our virtual navigation task; however, these measures may not have been sufficient to detect other changes in cognitive processing such as strategy shifts [10, 11]. This is important because more subtle aspects of cognition or memory may also be influenced by psychosocial stress. Furthermore, our virtual navigation task involved only visual sensory input in contrast to a combination of multiple systems such as visual, kinesthetic, and vestibular input, which are available during immersive VR (e.g. head mounted display) or real navigation. To our knowledge, no studies have investigated whether stress differentially affects processing in these sensory subsystems, and none have directly assessed navigational performance while moving in large-scale space. Nonetheless, these systems may be important in typical spatial navigation; for example, inactivation of vestibular signals inhibits the firing of place cells and head direction cells in the hippocampus of rats, which are vital in spatial orientation [61].

Individual differences may have also played a role in our results. For example, the phase of the menstrual cycle was not taken into consideration as females were tested at all stages and oral contraceptive users (*n* = 13; 43% of females in our study) were not excluded. Previous work has shown that reactivity to laboratory challenges is affected by menstrual cycle hormonal fluctuation [62]. SAM activation in our study was measured through cardiovascular reactivity. Another indicator of SAM activation is the quantification of salivary alpha amylase, a neuroendocrine marker which plays an important role in the stress response [63]. Inclusion of alpha amylase for future research would provide a more direct measure of SAM activation. Finally, data collection sessions took place between 12pm and 5pm, but we did not have sufficient sample size to control for time of day in the statistical analyses.

Since SAM had diminished before learning in our study, another approach would be to examine the influence of stress immediately after or coincident with spatial performance, as central norepinephrine secretion and dopamine are released during stress and also modulate PFC functioning [64]. Future studies might investigate these relationships by employing behavioral studies used in conjunction with neuroimaging techniques (e.g. fMRI, PET, and rCBF) and physiological recording to tease out the effects of these different systems. More studies are needed to investigate the effects of stress on real world spatial challenges such as driving and navigating through traffic; especially since the UFOV spatial attention task is a predictor of driving ability [65].

## Conclusions

In conclusion, the present study contributes to our understanding of the influence of an acute laboratory stressor on two measures of spatial performance. Socially Evaluated Cold Pressor stress resulted in no change in speed or accuracy in a virtual navigation task. For the spatial attention task, the SECPT also led to male cortisol responders having better accuracy than females. Among females, higher stress-induced cortisol responses were also associated with lower spatial attention performance. HPA activation may be necessary to further understand sex differences in spatial performance and related to activity in the hippocampus and prefrontal cortex. These results may also be due to increases in SAM disruption in spatial memory retrieval through catecholamine release, a modulation of PFC functioning due to HPA activation and subsequent cortisol release, or a combination of these factors. These findings may be particularly relevant in stressful occupations such as air traffic controllers, first responders, and surgeons, where changes in attention or spatial memory may have significant consequences.

## Method

### Participants

Sixty-one non-smoking (31 males, 30 females) participants between the ages of 18–31 years (*M* = 19.48, SD = 2.22) were recruited through flyers and email announcements. Participants reported no chronic health problems, no use of prescribed medications that could affect physiological or mood responses, were not pregnant, and had a BMI between 19 and 30 (*M* = 24.20, SD = 2.64). Course extra credit or compensation ($15.00) was provided. The study was approved by the college’s Institutional Review Board and procedures were performed according to their guidelines and regulations. All participants provided written informed consent.

### Physiological measures

#### Impedance cardiography

Heart rate (HR) was measured with a HIC-3000 impedance cardiograph (Bio-Impedance Technology, Inc., Chapel Hill, NC), using three bipolar silver-silver electrodes and a tetrapolar band-electrode system in accordance with established guidelines [66]. Heart rate was acquired, processed, and scored with commercial software (COP_WIN/HR 6.10, Bio-Impedance Technology, Inc.).

#### Blood pressure

Systolic blood pressure (SBP) and diastolic blood pressure (DBP) were measured using an automated blood pressure monitor (SunTech Medical^®^ Tango Blood Pressure monitor, Morrisville, NC) at specific time points throughout the study (detailed below in the Procedure).

#### Salivary cortisol sampling

Four salivary cortisol samples were obtained using the oral swab method. Participants were asked to chew and saturate on a salivette made of synthetic cotton material for approximately two minutes. At the conclusion of collection, salivettes were inserted into a sterile polypropylene tube and then frozen at −80° C until assayed. Salimetrics Laboratory (State College, PA) performed the biochemical assay analysis in duplicate at the conclusion of the study. Inter- and intra-assay coefficients of variance were below 6%. The timing of the cortisol sampling (see Fig. 5) allowed for accurate measurement of peak salivary cortisol levels after stress exposure since levels peak around 30 min post stress [10, 43].

### Task descriptions

#### Socially evaluated cold pressor test (SECPT)

Participants submerged their dominant hand up to their wrist in ice water measuring 4° C for three minutes. The length of the socially evaluative cold pressor test is consistent with prior literature [27, 30]. Similar to the protocol developed by Schwabe et al. [31], a video camera with a bright light was positioned on the participants’ face and they were informed that their facial expressions would be recorded during the test; however, these instructions were deceptive as the video camera only served as a prop in order to add a socially evaluated component to elicit a stronger stress response. An experimenter remained in the room with the participant and feigned taking notes for facial expression analysis. The SECPT has been used to induce cortisol and blood pressure responses comparable to the commonly used Trier Social Stress Test [7, 10, 31].

#### Useful field of view (UFOV)

**Fig. 6:**
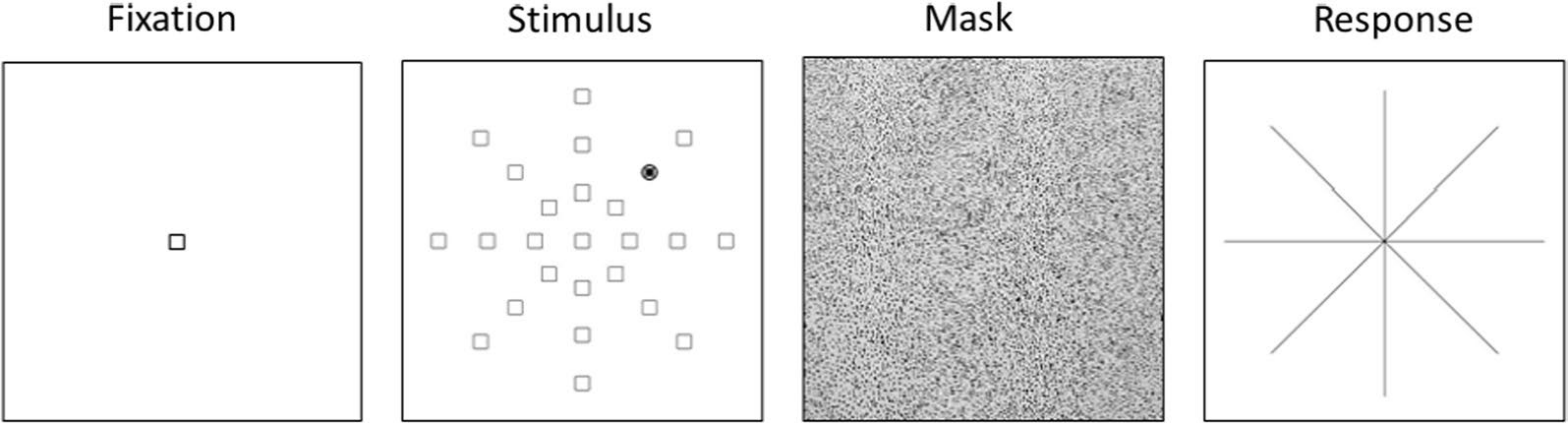
Sample Useful Field of View (UFOV) task. After fixation, a stimulus is presented for 5 ms followed by a mask. Participants then respond by indicating the location of the stimulus with a mouse

The UFOV is the area of the visual field that one can detect information without eye movement. The computer-based Useful Field of View task (UFOV) [67], which was modified and modeled after Feng et al. [46] served as our spatial attention task. Targets were presented rapidly and must be detected at different eccentricities from the center of the computer monitor. Specifically, a fixation square was presented for 600 msec to initiate each trial. Subsequently, the target stimulus, a dark square surrounded by a circle, was displayed for 5 msec at one of three eccentricities (10^o^, 20^o^, and 30^o^) from the center of the screen and along one of eight equally spaced radii (see Fig. 6). The stimulus was then replaced by a mask array for 600ms, and then a response cue was given in which participants indicated in which of the 8 directions the target had appeared. Participants were not given a time limit to respond and not given error feedback. Participants were positioned approximately 31.5 cm from the screen so that the visual angles were the same for all participants. After instructions and practice trials, participants judged two blocks of 32 randomized trials, indicating where the target flashed in terms of the eight possible directions for approximately ten minutes. Overall accuracy scores were then calculated as the number of correct responses.

#### Virtual reality navigation (VR)

**Fig. 7:**
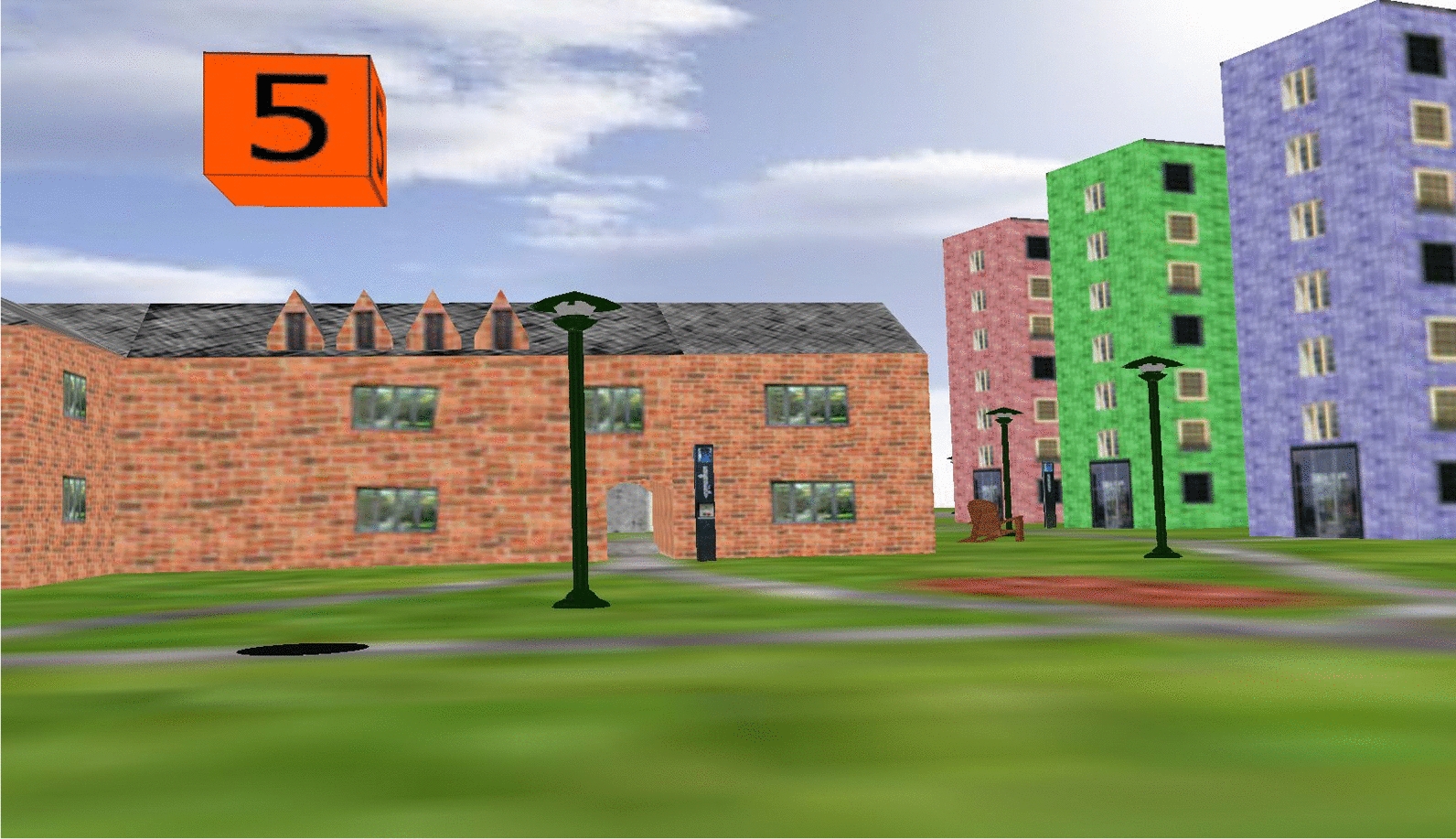
Sample environment used in the virtual navigation task

To assess spatial navigation performance, our second spatial task involved learning the location of targets in a virtual environment. The virtual environment was presented on a 19 inch LCD monitor using Vizard software (WorldViz, Santa Barbara, CA), and the task lasted approximately fifteen minutes. Using a joystick to navigate within the environments, seated participants learned the layout of four paths (two with 4 targets and two with 5 targets) in a virtual campus environment (see also [6]). Participants were verbally directed by the experimenter through a series of numbered targets and instructed to remember the locations of those targets along the path (see Fig. 7). Each path started at a target labeled 1, and continued with the sequence of numbered targets to the end of the path. After traversing the environment once, participants were returned to the beginning of the path (target 1) for testing. The experimenter asked participants to use the joystick to rotate their view to face in the direction of a non-visible target, and started a timer. When finished, participants were instructed to say “done” at which point the experimenter stopped the timer and proceeded with a second directional question. After traversing to the end of the path, participants made two additional directional judgments for a total of four judgments per path. Participants were also asked to rotate to face targets as quickly as possible, but not at the cost of accuracy.

Accuracy in the VR task was calculated as the absolute value of the deviation between the observed and correct response in degrees (absolute error). For example if the correct response to a target was 45 degrees, a 35 degree or 55 degree pointing response would both result in 10 degrees of pointing error. Pointing error was calculated by averaging their 20 pointing judgments. Participants were not given feedback about the accuracy of their judgments.

### Procedure

Participants were randomly assigned to either a Stress (*n* = 31; 16 males and 15 females) or Non-Stress condition (*n* = 30; 15 males and 15 females) prior to data collection. Testing occurred between 12 pm and 5 pm to control for circadian changes in salivary cortisol. All participants abstained from alcohol, caffeine, and strenuous physical activity for twelve hours and abstained from eating for four hours prior to the study session to control for short-term dietary or post-prandial effects [68, 69]. Before the experiment participants signed an informed consent form. After confirmation of normotensive status, height and weight were obtained to determine BMI, and bipolar silver-silver chloride electrodes and electrode band tape were appropriately placed on the participant for cardiovascular measurement. Participants were then seated and an inflatable cuff was applied to their non-dominant arm and connected to an automated blood pressure monitor.

Once participants were attached to the physiological recording equipment they were instructed to relax and listen to quiet music for a seated ten minute Baseline period. Next, participants in the Stress condition completed the three (see Fig. 5 for Timing of Measurements) minute Socially Evaluated Cold Pressor Test (SECPT) while the Non-Stress condition watched a 3-min relaxing undersea nature control Video. A ten minute resting recovery period (Recovery 1) in which participants listened to relaxing music followed both conditions. Immediately after the first recovery period, the ten minute Useful Field of View (UFOV) task and fifteen minute Virtual Reality navigation (VR) task were administered (as described above). The experiment concluded with a second ten minute resting recovery period (Recovery 2). Blood pressure was obtained every minute during the last three minutes of the Baseline period, every minute during the SECPT or Non-Stress period, and every other minute during the UFOV task, VR task, and both recovery periods. Impedance cardiography measurements were measured continuously throughout each task and period. Salivary cortisol samples were obtained at the conclusion of the Baseline period, after the UFOV task, after the VR task, and at the end of the second recovery period.

### Data reduction and statistical analysis

During the Baseline period, 55s ensemble averages were computed for HR; while SBP and DBP were averaged over the last three minutes. During the SECPT or control Video, UFOV, VR, and both Recovery periods, cardiovascular measurements were averaged across the entire task. Thus, cardiovascular reactivity was reduced to one value representing the Baseline period, SECPT or control Video, UFOV, VR, and both Recovery periods (1 and 2). Data were analyzed with analysis of variance (ANOVA) and Pearson correlational analysis. Greenhouse-Geisser corrections were applied when the sphericity assumption was not met [70]. A two-tailed alpha level of *p* <  0.05 was used to determine statistical significance for main effects and interactions for each analysis. For post-hoc tests, we applied Bonferroni adjustments to determine the significance of *p* values by dividing 0.05 by the number of comparisons. All data was analyzed using SPSS 28.0.

## Data Availability

The datasets used and/or analyzed during the current study are available from the corresponding author upon reasonable request.
